# A Miniaturized Nickel Oxide Thermistor via Aerosol Jet Technology

**DOI:** 10.3390/s17112602

**Published:** 2017-11-12

**Authors:** Chia Wang, Guan-Yi Hong, Kuan-Ming Li, Hong-Tsu Young

**Affiliations:** Department of Mechanical Engineering, National Taiwan University, Taipei 10617, Taiwan; D04522013@ntu.edu.tw (G.-Y.H.); kmli@ntu.edu.tw (K.-M.L.); hyoung@ntu.edu.tw (H.-T.Y.)

**Keywords:** printed thermistor, Aerosol Jet, nickel oxide, temperature sensing, printing technology

## Abstract

In this study, a miniaturized thermistor sensor was produced using the Aerosol Jet printing process for temperature sensing applications. A nickel oxide nanoparticle ink with a large temperature coefficient of resistance was fabricated. The thermistor was printed with a circular NiO thin film in between the two parallel silver conductive tracks on a cutting tool insert. The printed thermistor, which has an adjustable dimension with a submillimeter scale, operates over a range of 30–250 °C sensitively (B value of ~4310 K) without hysteretic effects. Moreover, the thermistor may be printed on a 3D surface through the Aerosol Jet printing process, which has increased capability for wide temperature-sensing applications.

## 1. Introduction

Temperature sensors are useful for sensing measurements in many dynamic applications, such as IC chips [1] and manufacturing processing [2]. Compared to commercial temperature sensors, printed sensors are more advantageous because they fit the surface topography of measurement situations, demand less space for installation, and may use a wider range of substrates. Using printing technologies allows for even more sensing applications, enabling sensors to be printed on flexible polyimide foil, injection molded devices, and PCB [3]. In addition, printed technology is easier to make temperature sensing arrays and is capable of measuring the temperature distribution of a system rather than its average temperature. Turning process is a machining process that is useful in many areas. Turning inserts are the cutting tools that turning processes use to remove material from workpieces [4]. Cutting temperature has been recognized as a major factor that directly affects tool life [5].

In the literature, some available methods have been discussed to showcase the possibilities of printed sensors. An inkjet-printed temperature sensor was made using commercial silver nanoparticle inks on paper as Ag resistance temperature detectors (RTDs) with dimensions of 16 mm × 16 mm [6], and it sensed temperatures between −20 and 60 °C with a temperature coefficient of resistance of 0.11%/K. An inkjet-printed nickel oxide thermistor array on a flexible substrate operated from room temperature to 200 °C with high sensitivity (B value of ~4300 K) [7]. Inkjet-printed square graphene thin films with dimensions of 8 mm × 8 mm on polyimide substrates were used for temperature sensing applications, and the sensing temperatures ranged from 25 to 175 °C with moderate sensitivity (B value of 1860 K) [8].

Aerosol Jet technology, a direct writing process, can deposit fine metal lines under 20 μm [9], utilizes nanoparticle inks in the range of 1–2500 cP, and can print polymers and adhesives. Conductor traces may be printed using gold, silver, or other nanoparticle inks [10], and uniform and continuous thin film patterns may be formed by depositing precise amounts of inks in specific areas at room temperature and atmospheric pressure [11].

**Figure 1:**
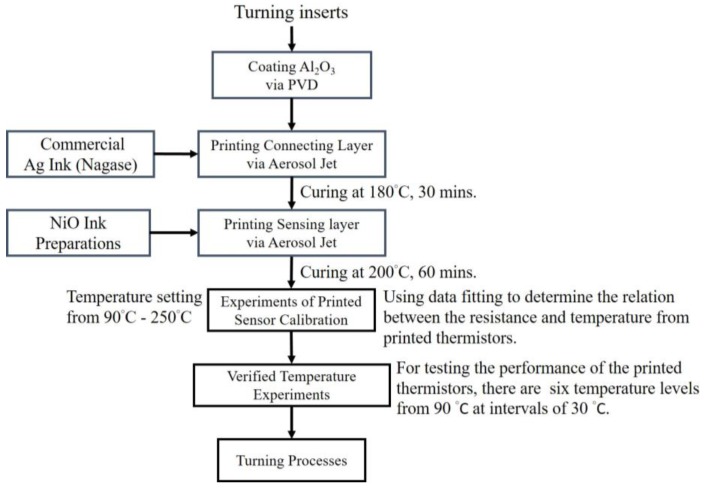
The research flowchart.

In Figure 1, the research flowchart shows how the printed thermistors were fabricated and confirmed through performance, and then used in real manufacturing and turning processes. In this study, the Aerosol Jet printing process was developed to fabricate miniaturized thermistors with high temperature sensitivity that can be printed directly on tiny cutting tools that are types of turning inserts. Nickel oxide is used as the temperature sensing material for printed thermistors because of its large temperature coefficient of resistance (TCR) and substantial chemical stability [7,12,13,14,15,16].

## 2. Experimental Setup

In order to print the thermistors on turning inserts, the insulation layer (Al_2_O_3_) is necessary. The curing temperature of NiO must be 200 °C for an hour to remove the ink solvents for NiO nanoparticle deposition [7].

**Figure 2:**
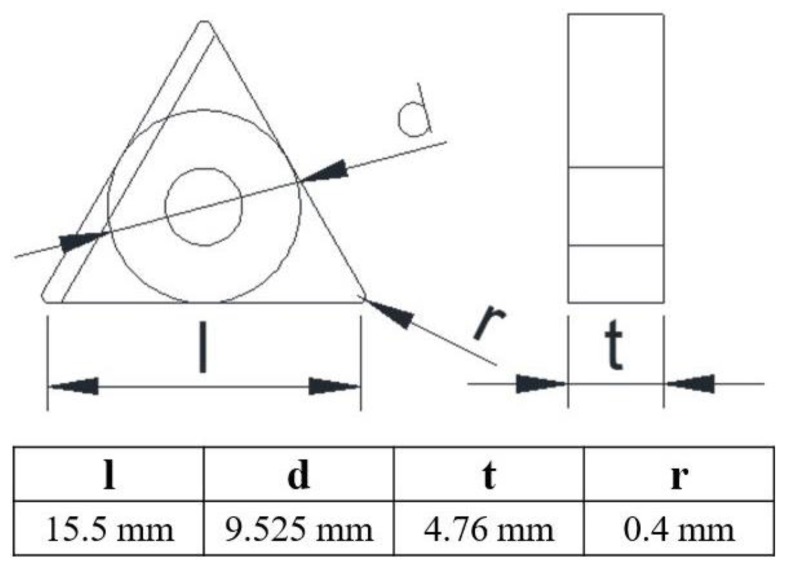
The dimensions of the turning inserts used in this study [4].

**Figure 3:**
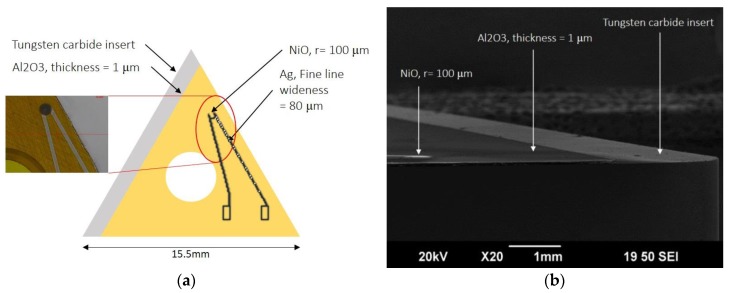
(**a**) The Al_2_O_3_ layer was coated on inserts for insulation; (**b**) the NiO thin film was examined by SEM.

The substrates used were cutting inserts for printed electronics (Figure 2), three different types of inserts that were chosen from KORLOY (Table 1) and have different 3D surfaces. In order to avoid the printed thermistors short-circuiting, all inserts were coated with an Al_2_O_3_ layer by physical vapor deposition (PVD) for insulation, and the thermistors were then printed on the Al_2_O_3_ layer (Figure 3). To further investigate the microstructure, the NiO thin film was examined by scanning electron microscopy (SEM). The dimension of each sensing element is about 0.1 mm (diameter), which is easy to produce through Aerosol Jet printing technology.

Silver nanoparticle ink (NAGASE, Osaka, Japan) was Aerosol Jet printed with a commercial continuous Optomec AJ-300 printer (Optomec, Albuquerque, NM, USA). The test pattern included 80 μm wide lines for both dimensional and electrical characterization. In this study, the silver conducting lines were deposited at 180 °C for 0.5 h, and NiO thin films were then deposited on them. Finally, the deposited silver conducting lines with NiO thin films were thermally calcined at 200 °C in a furnace for an hour [7] to remove solvents.

### 2.1. NiO Nanoparticle Ink Preparation

The fabricated method of NiO nanoparticle inks (Table 2) followed the literature [7]. A pH range between 2 and 8 is suggested [7,17] for suspension stability and chemical issues, so the pH value of NiO inks was controlled within a range of 3–5 in this study.

### 2.2. Aerosol Jet Printing Technology

**Figure 4:**
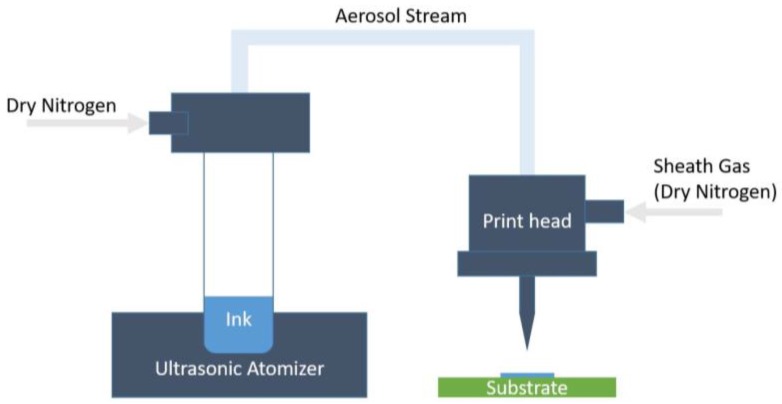
A schematic diagram of Aerosol Jet printing process.

Aerosol Jet technology was used to print Ag ink and NiO ink in this study. These liquid inks with dispersed nanoparticles were aerosolized using an ultrasonic atomizer. The diameter of the aerosolized droplets is 1–5 μm. These droplets flow to the print head, where they are surrounded by another gas, called the sheath gas, which aids collimating. N_2_ is used for both the carrier and sheath flows in this study. Finally, the focused aerosol flow, which is essentially a multi-phase stream of solid nanoparticles and ink solvents in a gas, is passed through a nozzle (100–300 μm in diameter) and deposited at an ejection speed of 100 mm/s on the target substrate (Figure 4).

### 2.3. Experiments with Printed Sensor Calibration

In this study, the resistivity versus the temperature characteristics were measured over a temperature range of 90–250 °C for at least three heating/cooling cycles. The printed thermistor of the *B* constant was calculated from the resistance versus the temperature characteristics using the Arrhenius Equation (1):

(1) $$B_{90/250}=\frac{\ln\frac{R_{90}}{R_{250}}}{\left(\frac{1}{T_{90}}\right)-\left(\frac{1}{T_{250}}\right)}$$

where *R*_90_ and *R*_250_ are the resistances measured at 90 °C (*T*_90_) and 250 °C (*T*_250_), respectively.

#### 2.3.1. Temperature Experiments via Heat Plate

**Figure 5:**
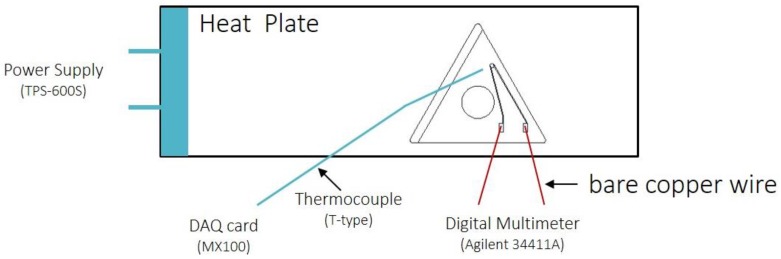
The experimental setup of the heating test.

The responses of the printed thermistors to temperature changes were evaluated through a heat plate experiment (Figure 5). The electrical resistance variation that resulted from the heat plate rose to 250 °C and then cooled down to 90 °C over the course of two cycles. The printed sensors were put on a heat plate first, and the hot plate, using a TPS-600S power supply, then elevated the temperature from 27 °C.

In order to confirm the capability of the printed thermistor, the commercial thermocouple and the printed thermistor were set to measure the temperature of the inserts in the same heating process (Table 3).

#### 2.3.2. Verified Temperature Experiments via Heat Plate

In order to test the performance of the printed thermistors, the heat plate was set to six different temperature levels, beginning at 90 °C and rising at intervals of 30 °C. The thermocouple was used to record the temperature and was compared with the printed thermistors in the same fashion, as previously described.

### 2.4. Turning Processes

**Figure 6:**
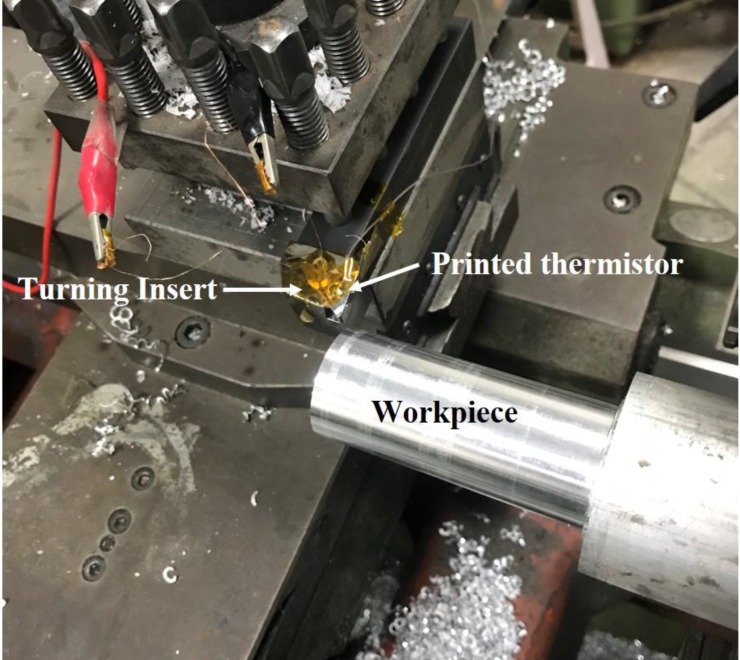
The turning processes were used to confirm the performance of the printed thermistor on the turning inserts.

Finally, the real turning processes were executed. There were three different turning depth levels that were tested in turning processes, and both the thermocouple and the printed thermistor were installed on the turning insert. The turning processes are shown in Table 4 and Figure 6.

## 3. Results and Discussion

**Figure 7:**
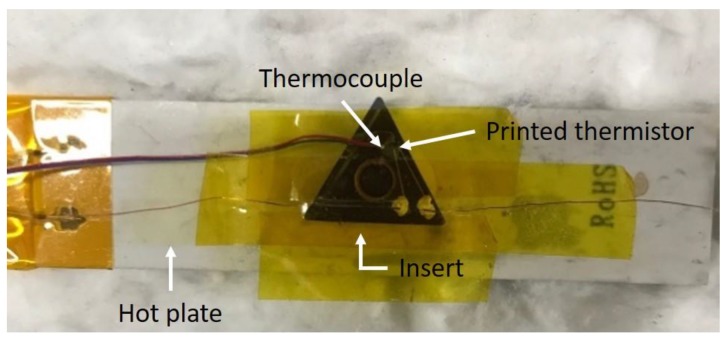
The experimental setup of the heat plate tests.

**Figure 8:**
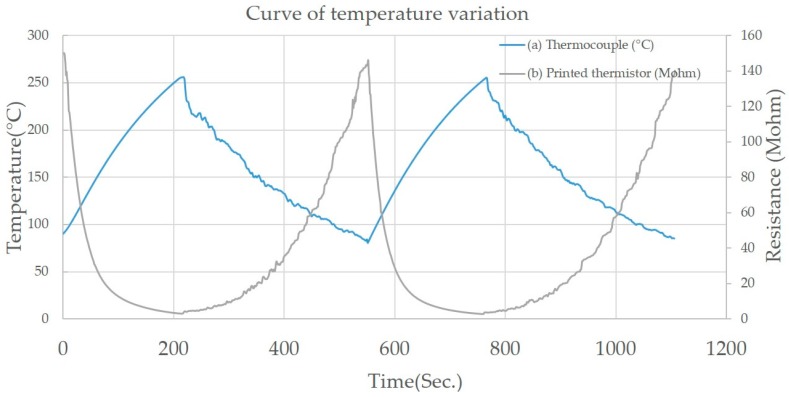
The two different temperature sensors in the heating process by heat plate: (**a**) the T-type thermocouple, and (**b**) the NiO-based printed thermistor on the turning insert.

The thermocouple was installed in close proximity to the printed thermistor (Figure 7), and it was found that both the thermocouple and the printed thermistor had the same approximate temperature during the heating process (Figure 8). Both the printed thermistors and thermocouple reacted in less than a second to a temperature change. This proves that the printed thermistor has a high sensitivity and is able to measure temperature changes with great precision.

### 3.1. The Results of the Temperature Experiments

**Figure 9:**
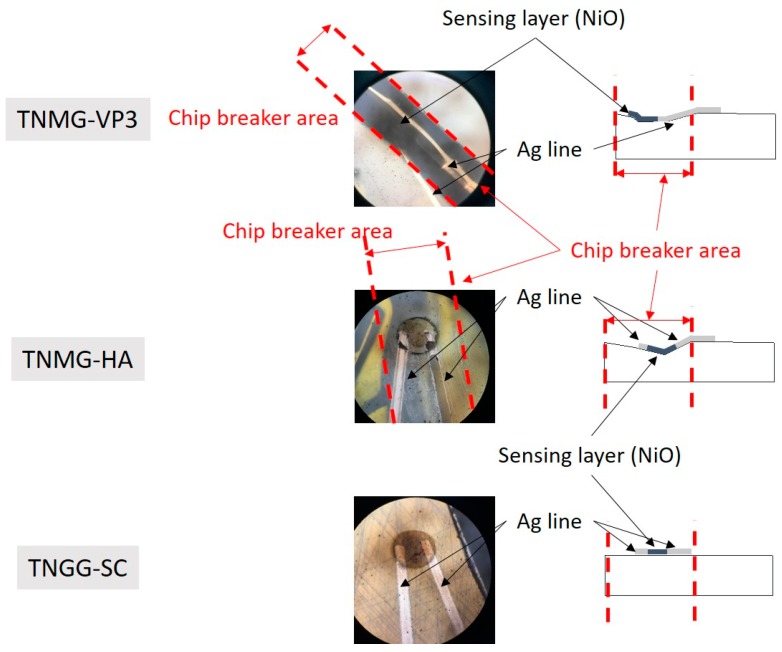
Three printed thermistors on different turning inserts [7].

**Figure 10:**
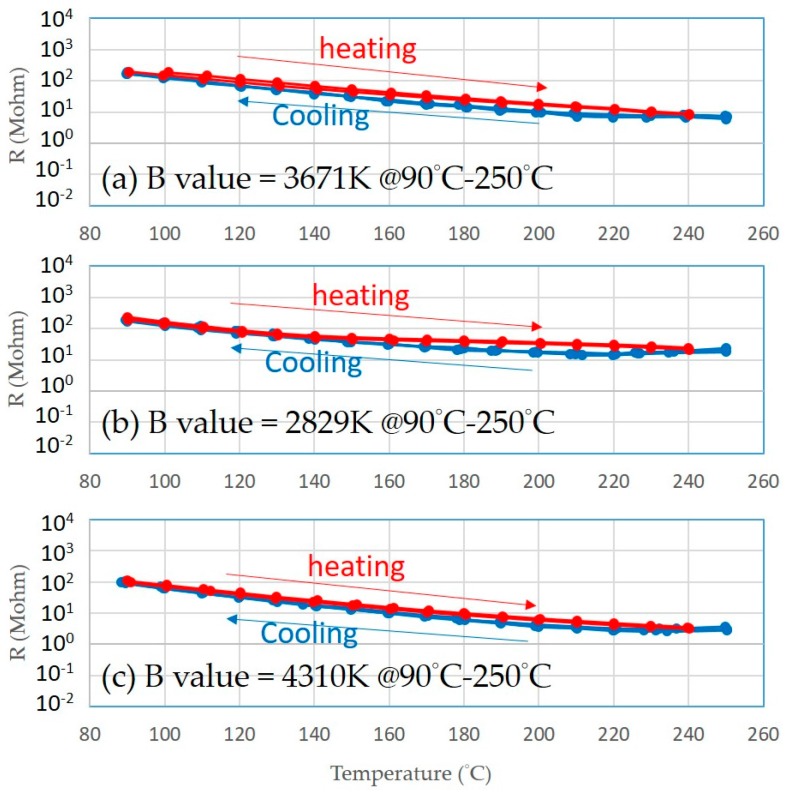
The printed thermistors on three different 3D surface inserts have stable curves in the heating process: (**a**) TNMG-VP3, (**b**) TNMG-HA, and (**c**) TNGG-SC.

The thermistors on three of the turning inserts (Figure 9) possessed great temperature sensitivity. Those printed thermistors were placed on the hot plate, and the transient profiles of electrical resistance with temperature variation were recorded (Figure 10). This showed that the B values of the thermistors went up to 4310 K (Table 5). This B value is close to that of conventional metal oxide negative temperature coefficient (NTC) materials, which are typically in the range of 2000 to 5000 K [13], and the heating and cooling cycles virtually overlapped, indicating that there was almost no electrical resistance hysteresis regarding temperature variation.

According to the resistivity variation of printed thermistors as a function of temperature, the relation was as shown in the equations below:

(2) $$T=458.69R^{-0.292}$$

(3) $$T=972.48R^{-0.462}$$

(4) $$T=332.76R^{-0.276}$$

where *T* is the temperature (°C), and *R* is the resistivity (Million-ohm). Equation (2) is the relation of TNMG-VP3, Equation (3) is the relation of TNMG-HA, and Equation (4) is the relation of TNGG-SC.

### 3.2. The Results of Verified Temperature Experiments

Finally, using Equations (2)–(4), three types of printed thermistors can be compared to commercial thermocouples in verified temperature tests.

**Figure 11:**
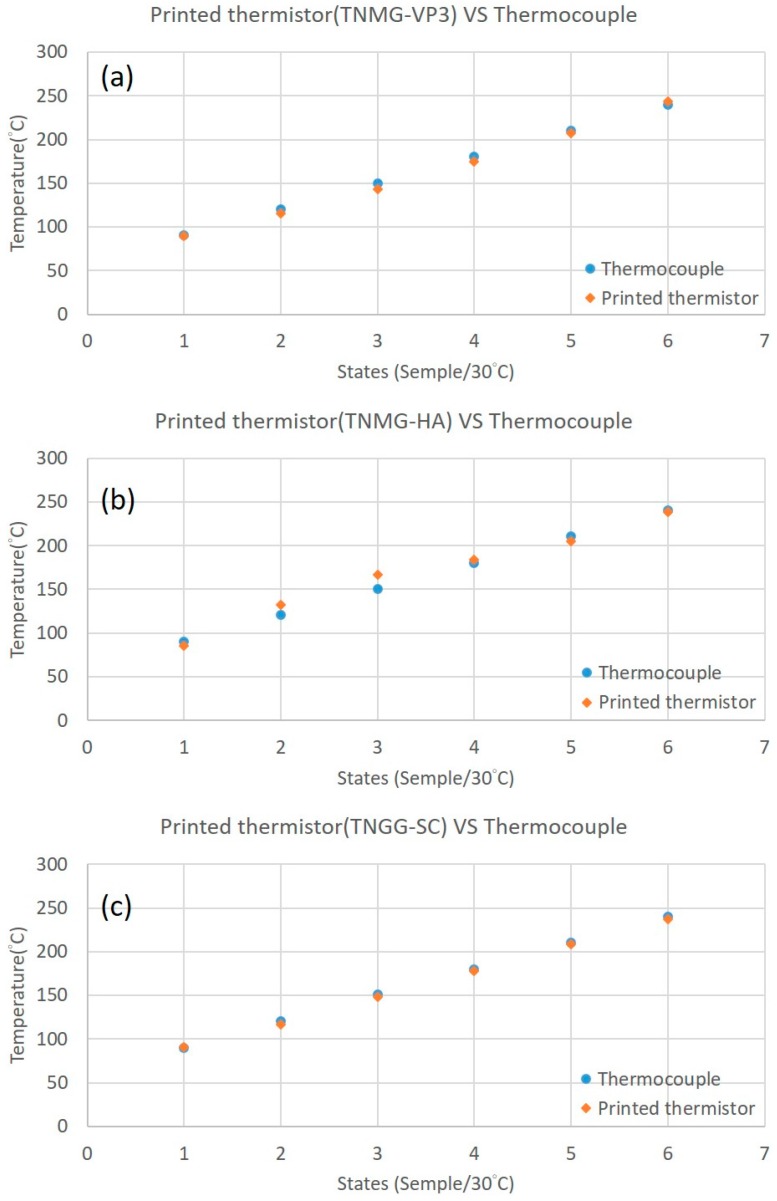
Verified temperature test results of three types of turning inserts. (**a**) TNMG-VP3, (**b**) TNMG-HA, and (**c**) TNGG-SC.

In the verified temperature tests, the temperature was measured by the commercial thermocouple, and the resulting resistance data of the printed thermistors was translated into temperature results by Equations (2)–(4). The printed thermistor showed a similar equilibrium temperature scale as that of the thermocouple (Figure 11).

Table 6 shows the temperature error percentage of the printed thermistor with thermocouple results. TNMG-HA has the largest average error percentage and TNGG-SC has the smallest average error percentage proportional to the complexity of the geometric shape.

### 3.3. Turning Process Results

**Figure 12:**
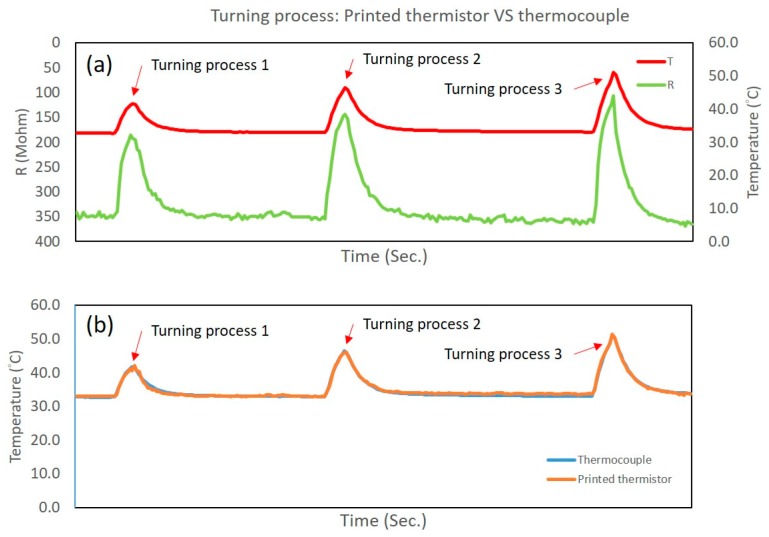
(**a**) The resistance of the printed thermistor and the measured temperature of the thermocouple. (**b**) The temperature estimated from the resistance of the printed thermistors; the two temperature curves are very close.

In the turning process results, the temperature that was measured by the thermocouple has an approximate variety and sensitivity according to the resistance that was measured by the printed thermistors (Figure 12). Using Equation (4), the resistance can be translated into temperature. The largest temperature error is under 3.2% during Turning Process 1. The standard deviation is 0.85%, and the average error is 0.3% during all turning processes. The two temperature curves are very close, illustrating the positive correlation between the thermocouple and the printed thermistors. The performance of the printed thermistors was confirmed in the turning processes.

## 4. Conclusions

The NiO-based 3D printed thermistors created via the Aerosol Jet printing process on turning inserts for temperature-sensing applications were shown to be highly capable. The printed thermistors on the three 3D inserts have high temperature sensitivity (B values of ~4310 K) without hysteretic effects, and the response time of the printed thermistors was almost as fast as that of the thermocouples. The Aerosol Jet technology has the flexibility to print circuit layouts on 3D structures, the thermistors may be allocated to different positions easily with a variety of circuit layouts, and the dimensions of the sensing element may be easily reduced below 0.1 mm. In summary, this study shows the high potential applicability of the sensors in the printed electronics area and provides an answer to the miniaturized sensing devices problem that is not limited by traditional coating/etching processes.

## Figures and Tables

**Table 1:** The four different turning types inserts [4].

Label	Turning Type	Feed Rate (mm/rev)	Depth of Cut (mm)
TNMG-VP3	medium	0.05–0.3	0.1–3.0
TNMG-HA	medium-finishing	0.05–0.3	0.8–3.5
TNGG-SC	finishing	0.05–0.25	0.3–2.0

**Table 2:** The ingredients of NiO nanoparticle inks [7].

Material	Weight (g)
Propylene Glycol Methyl Ether	2
Deionized water	8
Nickel oxide powder	0.5

**Table 3:** The measured equipment for two different temperature-sensing sources.

Equipment	Model	Sensitivity	Signal Source	Source Type
Digital Multimeter	Agilent 34411A	1 Sample/s	Printed thermistor	Resistance
DAQ card	MX100	1 Sample/s	Thermocouple	Temperature

**Table 4:** The experimental setup of the turning processes.

Workpiece Material	Workpiece Size	Turning Insert	Process Feed	Process Depth	Process Speed
Aluminum	60 mm (D) × 750 mm (L)	TNGG-SC	0.105 mm/rev	1/1.5/2 mm	1800 rpm

**Table 5:** The corresponding B values for the printed thermistors.

Turning Insert Type	B Values (90 °C–250 °C)
(a) TNMG-VP3	3671 K
(b) TNMG-HA	2829 K
(c) TNGG-SC	4310 K

**Table 6:** The temperature error percentage of each printed thermistor.

States	(a) TNMG-VP3	(b) TNMG-HA	(c) TNGG-SC
1 (90 °C)	−0.1%	−5.2%	1%
2 (120 °C)	−4.2%	9.6%	−2.8%
3 (150 °C)	−4.3%	10.7%	−2.4%
4 (180 °C)	−3.1%	1.8%	−1.3%
5 (210 °C)	−1.3%	−2.7%	−1%
6 (240 °C)	1.5%	−0.8%	−1.2%
